# A Survey Aimed at General Citizens of the US and Japan about Their Attitudes toward Electronic Medical Data Handling

**DOI:** 10.3390/ijerph110504572

**Published:** 2014-04-25

**Authors:** Michio Kimura, Jun Nakaya, Hiroshi Watanabe, Toshiro Shimizu, Kazuyuki Nakayasu

**Affiliations:** 1 Department of Medical Informatics, School of Medicine, Hamamatsu University, Hamamatsu 431-3192, Japan; 2 Medical IT Center, School of Medicine, Tohoku University, Sendai 980-8574, Japan; E-Mail: junnaka@med.tohoku.ac.jp; 3 Department of Clinical Research and Development, National Center for Geriatrics and Gerontology, Ohbu 474-8511, Japan; E-Mail: hiroshiw@ncgg.go.jp; 4 SBS Information Systems, Shizuoka 422-8033, Japan; E-Mail: t_shimizu@sbs-infosys.co.jp; 5 Graduate School of Health Sciences, Hokkaido University, Sapporo 060-8638, Japan; E-Mail: nakayasu-cazzyuki@hs.hokudai.ac.jp

**Keywords:** surveys, public opinion, electronic health records, privacy, internet

## Abstract

*Objectives*: To clarify the views of the general population of two countries (US and Japan), concerning the handling of their medical records electronically. *Methods*: We contacted people nationwide in the United States at random via Random Digit Dialing (RDD) to obtain 200 eligible responders. The questionnaire was for obtaining the information on their attitudes towards handling of their medical records, disclosure of the name of disease, secondary usage of information, compiling their records into a lifelong medical record, and access to their medical records on the Internet. We had also surveyed people of Shizuoka prefecture in Japan using same questionnaires sent by mail, for which we obtained 457 valid answers. *Results*: Even in an unidentifiable manner, US people feel profit-oriented usage of medical data without specific consent is not acceptable. There is a significant difference between usage of unidentifiable medical data for profit (about 50% feel negatively) and for official/research purposes (about 30% feel negatively). About 60% of the US responders have a negative view on the proposal that unidentifiable medical information be utilized for profit by private companies to attain healthcare cost savings. As regards compiling a lifelong medical record, positive answers and negative answers are almost equally divided in the US (46% *vs*. 38%) while more positive attitudes are seen in Japan (74% *vs*. 12%). However, any incentive measures aimed at changing attitudes to such a compiling including the discount of healthcare costs or insurance fees are unwelcomed by people regardless of their age or health condition in both surveys. Regarding the access to their own medical record via the Internet, 38% of the US responders feel this is unacceptable while 50.5% were willing to accept it. *Conclusions*: Participants from the US think that the extent of the sharing their identifiable medical records should be limited to the doctors-in-charge and specified doctors referred to by their own doctors. On the other hand, Japanese people find it acceptable for doctors of the same hospital to share their medical records. Even in unidentifiable manner, people in both countries think the profits resulting from the secondary use of medical records should be returned to the public or patients. With regard to compiling a lifelong medical record, participants from the US provided both positive answers and negative answers, while more positive attitudes were observed in Japan. However, any incentives or measures aimed at changing attitudes towards such a compilation, including provision of a discount on healthcare costs or insurance fees, were not welcomed by participants from US as well as those from Japan, regardless of their age or health condition.

## 1. Introduction

Information technology has promoted collaboration and specialization within community healthcare networks [1,2], while many countries have initiated Electronic Health Record (EHR) projects [3,4,5]. Already, evaluation of applications of EHR to healthcare and research has been reported [6]. Under such circumstances, patients’ medical data is transferred and handled without using paper or CD-ROMs. We conducted a questionnaire survey to investigate the awareness of people regarding medical data handling in this manner. In the present study, it was hypothesized that the sharing medical data among different healthcare providers and compiling them into one life-long record, supported by secondary use of anonymous data via internet, is not yet accepted in these countries.

The target population for this survey was not physicians or patients, but the general population. Similar surveys have been conducted with physicians [7,8,9,10,11] and patients [12,13,14,15,16,17], but few studies have targeted citizens [18,19,20,21]. As public funding allocated to healthcare services is expected to expand, taxpayers’ views should be of significant concern as well as the opinions of patients and healthcare personnel. The general population includes healthy people, people that are, and had been in therapy.

The research questions are:

(1) What kinds of doctors, public organizations, private companies, people allow to access their medical data?
(2) Is a scheme allowing healthcare providers or private companies to access people’s anonymous medical data for the purpose of healthcare cost savings accepted?
(3) Do people prefer to compile their medical records into one life-long record?
(4) Is access to their medical data via the internet considered acceptable and safe?
(5) Do the results differ between these two countries, considering the differences in their healthcare policy?

## 2. Methods

### 2.1. Survey in the US

The target of this survey was ordinary citizens who live in the US. On 28 September 2009, we contacted people at random via US nationwide Random Digit Dialing (RDD) to obtain 200 eligible responders aged 19 years old and over who completed the telephone interview. 

### 2.2. Survey in Japan

A survey using the same questions was conducted in Japan a year before the above U.S survey. The target was the general population including men and women aged 20 to 69 years old who live in Shizuoka Prefecture (population in 2010 was 3,760,000, about 1/35 of that of all Japan). The questionnaire was sent to 2,000 households which were selected at random from the telephone directory. We asked that the responder should be a person whose birthday was nearest to the received date among the family members aged 20–69 so that we could obtain responses from different age groups. This was done because without this assignment, the elderly are more likely to become responders because they are likely to stay at home. The survey period was 16–31 October 2007. 

### 2.3. Statistical Analysis

The non-parametric analysis procedure, Pearson’s chi-square test, was used to compare the participants’ responses. 

### 2.4. Explained Definition of “Identified” and “Unidentified”

About the terms “identifiable” and “unidentifiable,” we consciously use these terms with only some explanation in both surveys due to limited response time, although we supposed that there may be different level of understanding among people. There are many methods of making information de-identified [22,23,24,25], the explained definition of “identifiable” was “ with your name and address,” and of “unidentifiable” was “ without your name, address, your other access, and your clinical history, is made anonymous such that nobody can spot you.”

### 2.5. Questionnaires

See Appendix.

## 3. Results

### 3.1. Responder Attributes

The cooperation rate was calculated based on the definition of the American Association for Public Opinion Research (AAPOR) [26]. Each case was coded according to one of the AAPOR categories. These categories were as follows: 

US survey: I (Completed Interviews) = 200, P (Partial Interviews) = 28, R (Eligible, Non-interview, Refusal) = 443, NC (Eligible, Non-interview, No Contact) = 8,649, O (Eligible, Non-interview, Other) = 81, UH (Unknown Eligibility) = 10,141, and NE (Not Eligible) = 1,367. The Cooperation Rate (AAPOR CR4) was calculated by employing the formula: CR4 = (I + P)/(I + R + P). The cooperation rate of this survey was 34.0%.

Japan survey: I = 457, P = 53, UH (Unknown Household) = 29 and UO (Unknown Other) = 1,340. The Response Rate (AAPOR RR4) was calculated by employing the formula: RR2 = (I + P)/(I + P) + (R + NC + O) + (UH + UO), and the response rate of this survey was 25.5%.

The average session period, for the US survey, was 23 min 25 s.

The attributes of eligible respondents in the US and the Japan survey have been summarized in the following Table 1.

**Table 1:** Respondent attributes.

Categories	Attributes	US	Japan
Sex	Male	42.5%	76.1%
	Female	57.5%	23.9%
Age	19–29	8.0%	2.9%
	30–39	12.0%	5.7%
	40–49	11.5%	15.7%
	50–59	27.0%	33.1%
	60–69	22.5%	42.5%
	70+	17.5%	
	No response	1.5%	n/a
Current physical condition	Healthy	60.0%	30.0%
	Rather healthy	25.5%	49.2%
	Not very healthy	9.0%	16.8%
	Not healthy	5.5%	4.0%

### 3.2. Questionnaire Results

**Figure 1:**
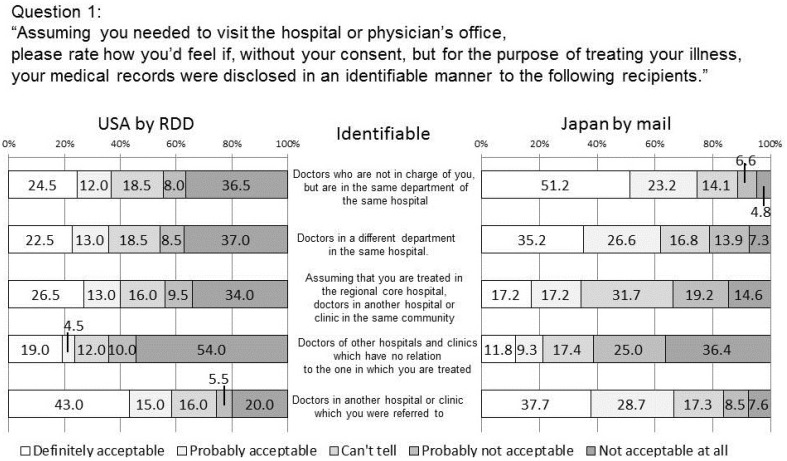
How would you feel if, without your consent, your medical records were disclosed to these doctors/organizations in an identifiable manner?

**Figure 2:**
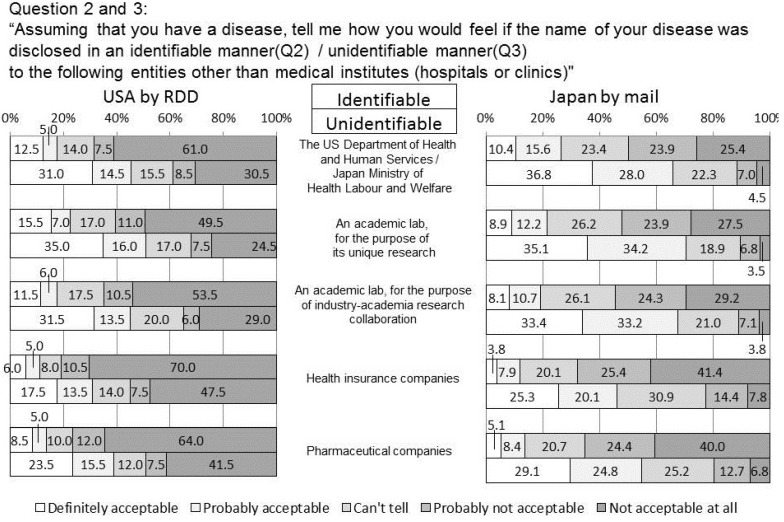
How would you feel if, without your consent, the name of your disease was disclosed to these organizations in an identifiable/unidentifiable manner?

**Figure 3:**
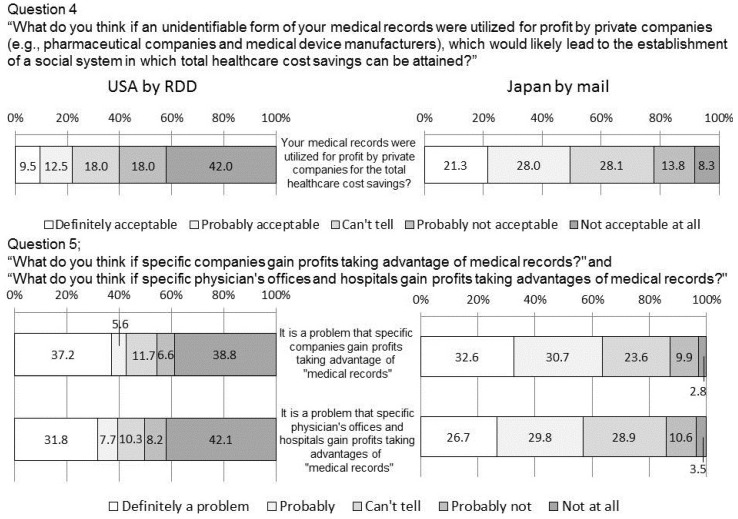
Findings related to secondary usage of information.

**Figure 4:**
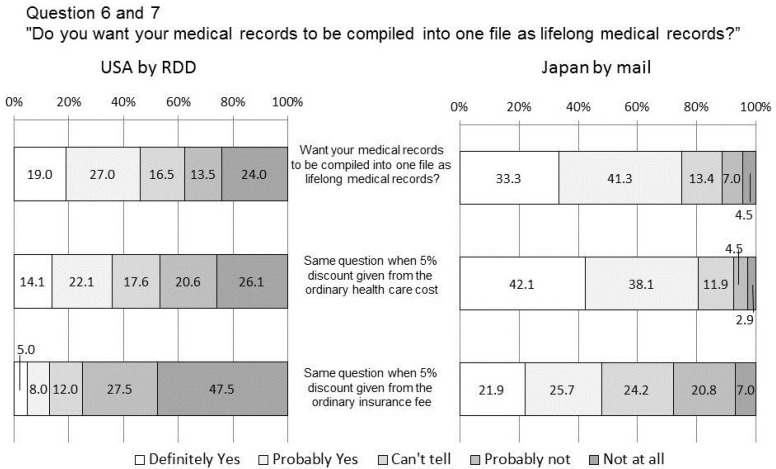
Do you want your medical records to be compiled into one file as lifelong medical records? How about with 5% off on medical cost? with 5% insurance discount?

**Figure 5:**
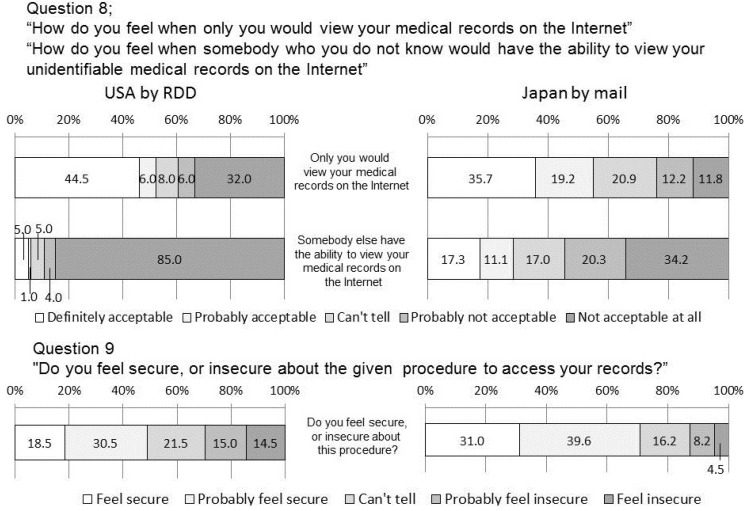
Findings related to access to medical records on the internet.

The questionnaire results have been presented in the following Figure 1, Figure 2, Figure 3, Figure 4 and Figure 5 and Table 2.

**Table 2:** Expected benefits of healthcare IT innovation (in the US survey only) explored by the item, “Please let me know which, if any, of the following you would expect to be achieved by the digitization of medical records and accessing these records online?” The responders were asked to choose one of the following options.

Percentage	Single choice answers
21.0%	Being able to receive treatment of the same quality at any physician’s office or hospital
18.0%	Avoiding duplicated tests and prescriptions
9.0%	Healthcare cost savings
5.0%	Receiving an adequate explanation for your disease
4.0%	Promotion of collaborative community healthcare
4.0%	Provision of a lifelong medical record system
4.0%	Contribution to future medical progress
1.5%	Access to health care at a nearby hospital
1.0%	Establishment of a team-based healthcare environment in various medical facilities
32.5%	Other

## 4. Discussion

### 4.1. Q1 about the Handling of Medical Records

In the US survey, in the identifiable situation, except for the case with referral doctors, almost half or more US people have a negative attitude defined as a cumulative response of “Probably not acceptable” and “Not acceptable at all” (hereafter referred to as negative). This indicates that sharing of their medical records without consent to doctors other than the doctors in charge or a referral doctor is not acceptable.

There is a significant difference (*p* < 0.01) between “referred doctors” (negative: 25.5%) and “doctors not in charge but in the same department of the same hospital” (44.5%) whereas no significant difference is seen between “doctors in the same department of the same hospital” and “doctors in a different department of the same hospital”. There is a barrier between people’s attitudes to “referred doctors” and “doctors in the same department of the same hospital”

Note that there is a significant difference between “referred doctors” and “doctors in the same community,” which indicates people think that the range of the medical record sharing should be limited to referral doctors determined by their own doctors and think it should not be assigned regionally. In a questionnaire survey conducted in five clinics in Australia and New Zealand [13], patients’ attitudes toward sharing their electronic health records (EHR) were found to be influenced by three factors which were identity of recipient: level of anonymity: and type of information: In this survey, we obtained similar results.

As for the survey conducted in Japan which is shown to the right of Figure 1, negative attitude is significantly less than that of the US survey for all items.

In the Japanese survey, there is no significant difference between “referred doctors (negative 16.1%)” and ”doctors in the same department of the same hospital (negative 11.4%)” whereas a significant difference (*p* < 0.01) is observed between each of those two recipients and doctors in a different department of the same hospital (21.2%). This indicates that the Japanese participants had a tendency to believe, “I am treated in the department of the hospital,” whereas Americans had a tendency to believe, “I am treated by the doctor.”

As for any difference between men and women (not shown in the figures), for example, in the US there is no significant difference between men (48.2%) and women (44.4%) who have negative attitudes toward the sharing of their information with a regional core hospital whereas a significant difference (*p* < 0.01) is seen between Japanese men (28.3%) and women (39.2%) who answered negatively to the same question.

### 4.2. Q2 and 3 Disclosure of the Name of the Disease

Although notifiable infectious diseases must be reported, more than half the US responders felt negative not only about sharing just the name of the disease in an identifiable manner for profit-oriented research as a matter of course, but also for official and non-profit purposes. When the usage of information contains both the individual’s name and the name of the disease, it is a strict requirement that it should be for the public good and under the control (such as punitive measures taken against those allowing an information leak) of a reliable administrator.

In information given in an unidentifiable form, 39.0%, 32.0%, 35.0% of the US responders answered negatively for the public/research purposes and 55.0% and 59.0% for commercial usages, showing a significant difference (*p* < 0.05) between any pairs, one each from the two groups. However, no significant difference was observed in the negative attitudes within the public, academic, and industry-academia research collaboration.

In Japan, 11.5%, 10.3%, 10.9% of the responders have negative attitudes to information usage for the purposes of public interest and 22.2%, 19.5% for the purposes for profit of business respectively. As long as there is such a substantial negative attitude in both countries, an opt-out consent approach is not considered to be acceptable, not only in the US, but also in Japan. In other words, an opt-in approach should be required when considering the possibility of commercial secondary usage. As indicated in a study about possible forms of consent in an electronic environment [10], blanket consent cannot always serve the needs of each subject and the content of consent should be designed on a case-by-case basis, although this could be time consuming.

### 4.3. Q4 and 5 Secondary Usage of Information

Figure 3 shows people’s attitudes toward the proposal that patients would provide their medical records in unidentifiable form to a profit-oriented private company for attaining healthcare cost savings. To this, 22.0% of the US responders have favorable attitudes and 60.0% have negative attitudes. Willison’s survey [19] of the Canadian public, concerning consent of secondary use of unidentified data in 2007 showed that 11% felt no need for notification or consent, 24% supported notification and opt-out, while 32% needed consent for each use. 22% favorable attitude of our study is considered almost similar to sum of 11 and 24 of the Canadian survey. 

The last two segments of Figure 3 show the results of the question on how they would feel if specific companies or hospitals gained profits from such a business model. About 40% of the US responders and more of the Japanese responders answer negatively, suggesting people in both countries think the profits should be returned to the public or patients

### 4.4. Q6 and 7 Lifelong Medical Records

In this survey, Electronic Medical Record (EMR) refers to the individual lifelong medical electronic records. Based on this definition, we presented both the advantages and disadvantages briefly and then asked whether respondents wanted their medical records to be compiled into one (without asking how they would be used). As shown in Figure 4, in the US survey, positive answers were 46.0% and negative answers were 37.5%, which yielded no significant difference. In Japan, positive answers were 74.6% and negative answers were 11.5%. The US answer of 46.0% positive was lower than expected. This could be because the questionnaire wording “compiled in one file” caused more fear of privacy risk.

Hoerbst’s survey for EHR among Austrian and German citizens [20] showed that between 80% and 90% were supportive of the idea of exchanging health related data between health care providers. Also, Perera’s survey citizens of Ontario, Canada [21] showed that most (>90%) supported the computerized sharing of the patient’s health record among their health care providers. Thinking that our questionnaire is clearly stating “compling as one file”, these Austrian/German and Ontario answers are nearer to Japanese 74.6% positive answers.

When asked whether they were interested in compiling their medical records into one lifelong medical record if they were given a 5% discount from the ordinary health care cost as a result, US negative answers increased (46.7%) the difference of which was however not significant. However, when asked whether they would be interested if they were to receive a 5% discount from the ordinary insurance fee if their lifelong medical records were disclosed, US positive answers further dropped off and negative answers significantly increased to 75.0% (*p* < 0.01). No significant differences between answers of healthy (negative 74.9%) and non-healthy (75.9%) responders and between answers of people aged 39 and under (67.5%) and those aged 40 and over (78.9%). This suggests such discount incentives do not have much efficacy.

In Japan, favorable attitudes towards savings of healthcare cost are relatively higher but people react negatively to the suggestion of an insurance fee discount.

Little change is observed in attitudes of the responders either in the US or Japan when proposed healthcare cost savings. The result, however, indicates that people in both countries may fear the possibilities of cherry picking by insurance companies (trying to contract only with low risk people). In the US, people can choose insurance, and at the same time, insurance companies can choose the people to whom they offer the policy. This discount is thought to be an invitation for people with lower health risks, while those with high risk may lose a chance to avail a moderate price. In Japan however, healthcare insurance coverage is universal. This resulted in lesser change in the unfavorable response of the participants. This is in contrast to the perceived health condition of the respondents, as presented in Table 1 on respondent attributes. Note that 60.0% of the American respondents rated themselves as “Healthy,” while the same was rated by only 30.0% of the Japanese respondents. This may indicate that Japanese people have a tendency to avoid choosing extreme (definite) options, which is clear from the minor difference in the combined numbers of “Healthy” and “Rather healthy” (85.5% to 79.2%).

### 4.5. Q8 and 9 Access to Medical Records on the Internet

As regards to reference of their own medical records on the Internet, 50.5% of US responders answered favorably while 38.0% had negative attitudes. It is interesting to note that a substantial number of responders (44.5%) answered that they thought it was acceptable in a positive manner whilst a considerable number of the responders were negative. In order to serve the needs of people with a positive attitude at the same time as maintaining trust in the healthcare information system in relation to the people with negative attitudes, it would be desirable that only the data of those in agreement should be placed in access servers with outside access and that the data in such servers should be clearly distinguished from the database of the hospital information system.

Note that as many as 89.0% of the US responders are against access from unidentified people even in unidentifiable form. This suggests that the system in which anyone can have unlimited access to medical records as public property even in an unidentifiable form would lead to heavy criticism.

A study evaluated patients’ attitudes towards access to computerized patient records, which resulted that this may compromise safety [27]. This US-Japan survey aimed at citizens revealed the pros and cons are still weighted equally about the public confidence in the internet communication of medical records in technical terms.

### 4.6. Q10 Expectation for Healthcare IT Innovation

We explored the respondents’ expectations regarding healthcare IT innovation, which was asked as a single-choice question (refer to Table 2). High expectations were placed on options such as avoiding duplicated tests or receiving above a certain level of healthcare, while low expectations were given for collaborative community healthcare, access to health care at a nearby hospital and provision of a lifelong medical record system. After all, the latter three expectations have been produced from the viewpoint of healthcare providers whereas the former two represents actual public opinions. We consider that the reason for the lumbering healthcare collaboration via IT could be attributed to the discrepancy between what are expected and what can be achieved.

The same options concerning expectation for healthcare innovation were used in the questionnaire conducted by the Fukuoka City Medical Association in Japan in 2002, targeting patients who participated in a regional network of electronic medical records project [28]. In the Japanese surveys, the responders were allowed to mark all that apply. When comparing the Japanese 2008 survey (which has been reported) (hereafter referred to as the Shizuoka survey) with the Fukuoka 2002 survey , the responders in both cities selected some options at similar rates including “receiving above a certain level of healthcare (Shizuoka: 61%, Fukuoka: 65%), “receiving an adequate explanation (Shizuoka: 59%, Fukuoka: 56%)” and “provision of a lifelong medical record system (Shizuoka: 28%, Fukuoka: 27%). On the other hand, Shizuoka showed a higher percentage for other options such as “avoiding duplicated tests and prescriptions (Shizuoka: 69%, Fukuoka: 56%)” and “access to health care at a nearby hospital (Shizuoka: 41%, Fukuoka: 32%)” while Fukuoka had a higher percentage in “promotion of collaborative healthcare (Shizuoka: 35%, Fukuoka: 55%)”. As regard to healthcare cost saving, 22% of the responders of the Fukuoka survey selected the option while 63% of the Shizuoka responders marked the option this time, which suggests a deteriorating medical situation has pervaded society during this period. The option “Contribution to future medical progress” was newly introduced in this survey, which accounted for a rather high percentage of 44%

### 4.7. Limitations of This Survey

The sample size (200 and 457 for US and Japan, respectively) was not very large to exhibit the phenomenon tested by the hypotheses sufficiently. Further, different methods were applied in the two surveys (RDD and by mail). As in Table 1, female responders were dominant over male in US survey, while most resopnders were male in Japan, though we requested answers by a family member whose birthday is the nearest. In addition, RDD is known to involve a significant level of bias [29].

Respondents of these surveys were living in their house, either contacted through the RDD in the US, or mail in Japan. Consequently, patients suffering from severe diseases may have been eliminated from this survey. Such patients may have a higher motivation to compile their medical records into one. 

The 2012 Commonwealth Fund survey [30] revealed that the percentage of doctors who used electronic patient medical records in their practice varied among countries. In this context, more than 90% of the doctors in the Netherlands, UK, Norway, New Zealand, and Australia used such electronic records, while the same was found to be 69% in the US. Japan has not joined this survey; however, a Japan Association of Healthcare Information System Industry survey in 2012 shows this figure to be 18.7% [31]. The surveyed countries were found to exhibit a low use of EMR as compared to other countries of Commonwealth survey. As a result, doctors are less accustomed to using digital medical records and the internet for healthcare. This is in contrast to the fact that that Taiwan started IC chip card identification for healthcare professionals since 2007 [5].

It is important to note the difference between the healthcare systems of the two countries surveyed in the present study. A universal coverage policy is maintained in Japan, while citizens have the choices to select their insurance provider (including none) in the US. Further, the consumption tax is rather low in these two countries (US less than 10%, Japan 5%), which is generally high, especially in the northern European countries, which cover healthcare mainly by tax budget. This may have affected the participants’ responses to Q6 and 7.

## 5. Conclusions

US people think that the range of the sharing of their identifiable medical records should be limited to the doctors in charge and specified referred doctors referred to by their own doctors. About 50% of the responders felt negatively about the sharing of medical data to other doctors of the hospital where they are treated or the regional core hospital. Japanese people think that their medical records may be shared by other doctors of the same hospital. More US responders have negative attitudes to information disclosure to unspecified healthcare institutes. This result highlights the importance of a clear indication of the identity of recipient in terms of public perception.

Even in an unidentifiable manner, US people feel profit-oriented usage of medical data without specific consent is not acceptable. There is a significant difference between usage of unidentifiable medical data for profit (about 50% feel negatively) and for official/research purposes (about 30% feel negatively). About 60% of the US responders have a negative view on the proposal that unidentifiable medical information is utilized for profit by private companies to attain healthcare cost savings.

As regards compiling a lifelong medical record, positive answers and negative answers are equally divided in the US, while more positive attitudes are seen in Japan. However, any incentive measures aimed at changing attitudes to such a compiling including the discount of healthcare costs or insurance fees are unwelcomed by both US and Japan people, regardless of their age or health condition.

Regarding the access to their own medical records via the Internet, 38% of the US responders feel this is unacceptable, while 50.5% were willing to accept it. On the other hand, there is strong opposition toward unlimited access to their medical records from unknown or unidentified people on the Internet even in unidentifiable form.

## Appendix

### Questionnaires

Q1. Assuming that you are needed to visit the hospital or physician’s office, please rate how you’d feel if, without your consent, but for the purpose of treating your illness, your medical records were disclosed in an identifiable manner to the following recipients?

For this item, respondents were asked to choose any one from the following options that best described his/her feelings: “Definitely acceptable”, “Probably acceptable”, “Can’t tell”, “Probably not acceptable” and “Not acceptable at all”.

The information regarding the recipient of the medical records was as follows: “Doctors who are not in charge of you, but are in the same department of the same hospital”, “Doctors in a different department in the same hospital”, “Doctors in another hospital which you were referred to”, “Assuming that you are treated in the regional core hospital, doctors in another hospital or clinic in the same community”.

Q2. Assuming that you have a disease, how would you feel if the name of your disease was disclosed in an identifiable manner to the following entities other than medical institutes (hospitals or clinics): the US Department of Health and Human Services (Japan Ministry of Health), an academic lab for the purpose of its unique research, an academic lab for the purpose of an industry-academia research collaboration, health insurance companies, and pharmaceutical companies?

The response options were the same as those for Q1.

Q3. The same question was asked again by replacing the expression “in an identifiable manner” by “in an unidentifiable manner”.

Q4. What would you think if an unidentifiable form of your medical records were utilized for profit by private companies (e.g., pharmaceutical companies and medical device manufacturers), which would likely lead to the establishment of a social system in which savings on total healthcare costs can be attained?

The respondents were not presented any specific model capable of bringing about cost by using the information.

The response options were the same as those for Q1.

Q5. What would you think about specific companies gaining profits by taking advantage of medical records? What would you think about specific physician’s offices and hospitals gaining profits by taking advantages of medical records?

The response options were: “Definitely a problem”, “Probably”, “Can’t tell”, “Probably not” and “Not at all”.

Q6. Lifelong medical records refer to individual medical records that include one’s lifelong history of diseases, medical care, and administration of drugs, all of which are compiled into one file, instead of being managed by each hospital or physician’s office. The advantages and disadvantages of lifelong medical records are as follows:

Advantage: Duplication of the same tests or drugs is avoided when you are treated by another physician.

Disadvantage: Your medical history cannot be concealed even if you do not want to disclose any part of it when you are treated by another physician.

Now, do you want your medical records to be compiled into one file as a lifelong medical record?

Q7. Would you be interested in compiling your medical records into one lifelong medical record if you were given a 5% discount on regular health care cost as a result? How would you respond if a special contract were proposed by a life insurance salesman saying that you would receive a 5% discount on the regular insurance fee if you showed him your lifelong medical records?

The response options for Q6 and 7 were “Definitely yes”, “Probably yes”, “Can’t tell”, “Probably not” and “Not at all”.

Q8. How would you feel if only you could view your medical records on the Internet? How would you feel when somebody else has the ability to view your unidentifiable medical records on the Internet?

The response options were the same as those for Q1.

Q9. Assuming that your doctor wouldn’t be allowed to see your lifelong medical records in his/her practice unless you present him/her the IC-enabled key card for your lifelong medical records and enter your PIN by yourself, would you feel secure or insecure about this procedure?

The response options were “Feel secure”, “Probably feel secure”, “Can’t tell”, “Probably feel insecure” and “Feel insecure”.

Q10. (Only in the US survey) Please let me know which if any of the following you would expect to be achieved by the digitization of medical records and accessing these records online?

The respondents were asked to choose any **one** of the following options:

- Being able to receive treatment of the same quality at any physician’s office or hospital
- Avoiding duplicated tests and prescriptions01
- Healthcare cost savings
- Receiving an adequate explanation for your disease
- Promotion of collaborative community healthcare
- Provision of a lifelong medical record system
- Contribution to future medical progress
- Access to health care at a nearby hospital
- Establishment of team-based healthcare environment in various medical facilities
- Other
